# Fixation-induced surgical segment’s high stiffness and the damage of posterior structures together trigger a higher risk of adjacent segment disease in patients with lumbar interbody fusion operations

**DOI:** 10.1186/s13018-023-03838-x

**Published:** 2023-05-19

**Authors:** Ping Cai, Zhieng Xi, Chao Deng, Jingchi Li, Xiaoyu Zhang, Yingguang Zhou

**Affiliations:** 1 Department of Orthopedics, Lianyungang Hospital of Traditional Chinese Medicine, Lianyungang, Jiangsu People’s Republic of China; 2 Department of Orthopedics, Affiliated Hospital of Nanjing University of Chinese Medicine, Nanjing, Jiangsu People’s Republic of China; 3 Department of Spine Surgery, Affiliated Hospital of Integrated Traditional Chinese and Western Medicine, Nanjing University of Chinese Medicine, Nanjing, Jiangsu People’s Republic of China

**Keywords:** Adjacent segment diseases, Finite element analysis, Lumbar interbody fusion, Biomechanical deterioration, Posterior structures

## Abstract

**Background:**

Adjacent segment disease (ASD) is a commonly reported complication after lumbar interbody fusion (LIF); changes in the mechanical environment play an essential role in the generation of ASD. Traditionally, fixation-induced high stiffness in the surgical segment was the main reason for ASD. However, with more attention paid to the biomechanical significance of posterior bony and soft structures, surgeons hypothesize that this factor may also play an important role in ASD.

**Methods:**

Oblique and posterior LIF operations have been simulated in this study. The stand-alone OLIF and OLIF fixed by bilateral pedicle screw (BPS) system have been simulated. The spinal process (the attachment point of cranial ligamentum complex) was excised in the PLIF model; the BPS system has also been used in the PLIF model. Stress values related to ASD have been computed under physiological body positions, including flexion, extension, bending, and axial rotations.

**Results:**

Compared to the stand-alone OLIF model, the OLIF model with BPS fixation suffers higher stress values under extension body position. However, there are no apparent differences under other loading conditions. Moreover, significant increases in stress values can be recorded in flexion and extension loading conditions in the PLIF model with posterior structures damage.

**Conclusions:**

Fixation-induced surgical segment’s high stiffness and the damage of posterior soft tissues together trigger a higher risk of ASD in patients with LIF operations. Optimizing BPS fixation methods and pedicle screw designs and reducing the range of posterior structures excision may be an effective method to reduce the risk of ASD.

## Introduction

Lumbar interbody fusion (LIF) is widely used in treating lumbar degenerative diseases (LDDs) [1, 2]. With different LIF operations widely used in the last two decades and the prolonged clinical follow-up period for LIF patients, surgeons paid more attention to related complications [3, 4]. The adjacent segment disease (ASD) has been widely reported in LIF patients.

The average incidence rate of ASD in LIF patients was 26.6% [5, 6], and potential clinical risk factors for ASD have been widely reported. Most risk factors can be explained well from the biomechanical perspective [7, 8]. Specifically, patients with longer LIF segments suffer a higher incidence rate of ASD; biomechanical research presents that the cranial segments suffer higher stress levels in models with longer fusion segments [9–12]. Meanwhile, patients with older age and original disc degeneration on the cranial segment suffer a higher risk of ASD, corresponding finite element analysis presents that the original disc degeneration in adjacent segments may aggravate postoperative biomechanical deterioration in adjacent segments [13, 14]. Therefore, biomechanical research could provide a reliable perspective to identify the potential risk factors for ASD in LIF patients [9, 15].

ASD caused by bilateral pedicle screw (BPS) fixation has been widely reported. Specifically, by inserting bilateral pedicle screws and two connecting rods, the instant postoperative surgical segment stability can be well constructed; this is significant for interbody osteogenesis [12, 16, 17]. However, the stiffness of titanium alloy is obviously higher than bony structures. Resulting surgical segment’s high stiffness caused by LIF operation and resulting biomechanical deterioration in adjacent segments play a prominent role in the ASD generation process [12, 18].

Recently, the biomechanical significance of soft tissues has also been reported. Studies proved that the posterior ligament complex (PLC) (consisting of the super spinal ligament (SSL) and inter-spinal ligament (ISL)) acts as a "tension band" in the flexion body position [19, 20]. The damage of PLC negatively affects the motion segment's stability and related local mechanical environment. Spinal process incision is a necessary surgical procedure in some LIF operations [21, 22]. Excision of the surgical segment's spinal process damages the attachment point of the cranial side PLC. This point may change the cranial motion segment's motility characteristics. Based on the above theoretical and practical foundations, we hypothesize that the fixation-induced surgical segment’s high stiffness and the damage of posterior structures together trigger a higher risk of adjacent segment disease in patients with LIF operations.

**Fig. 1:**
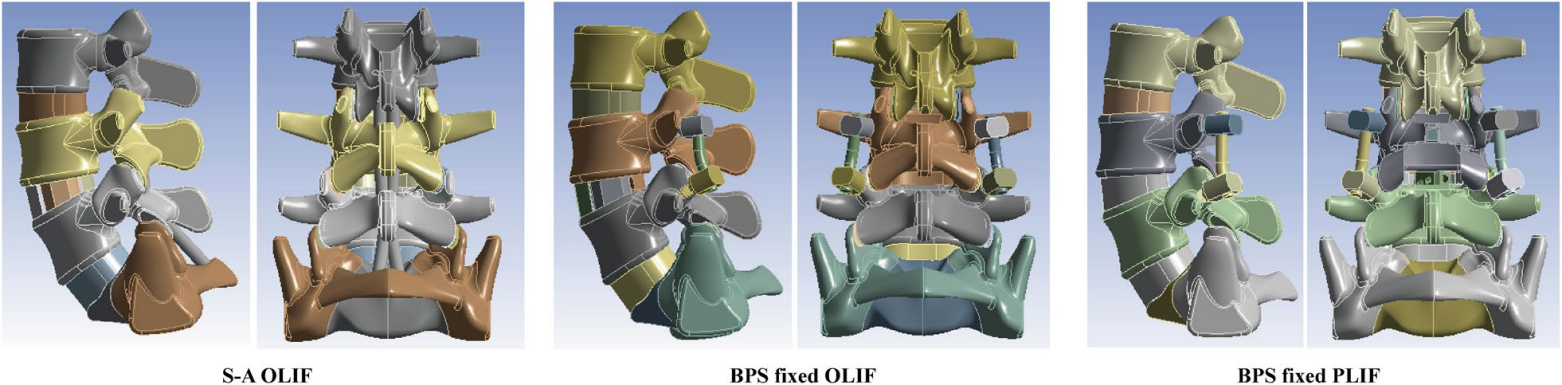
Simulation of LIF operations with and without BPS fixation and spinal process excision

**Fig. 2:**
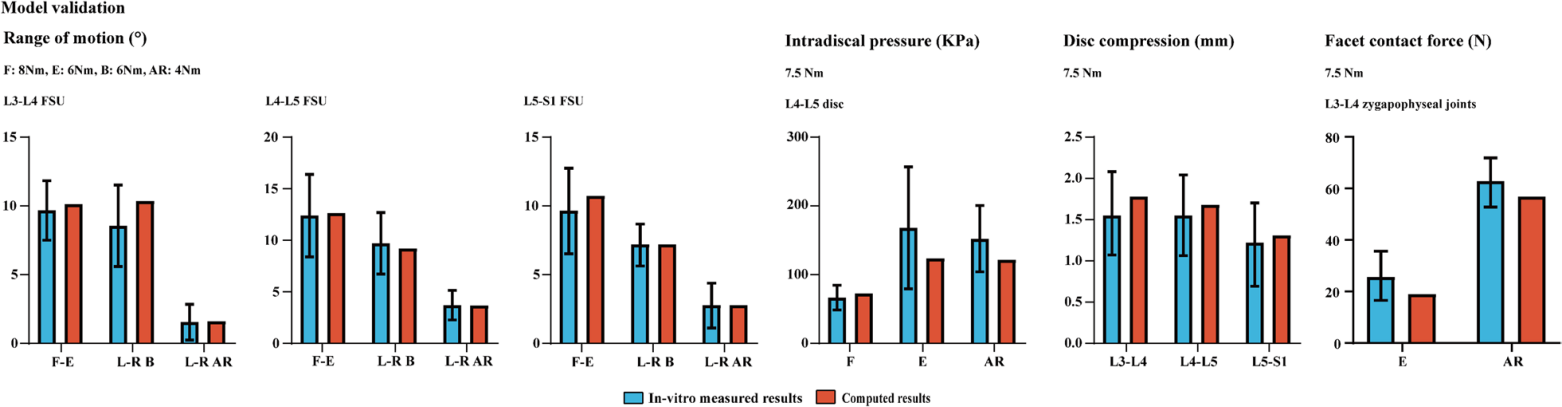
Multi-indicators model validation of the intact lumbo-sacral model

**Fig. 3:**
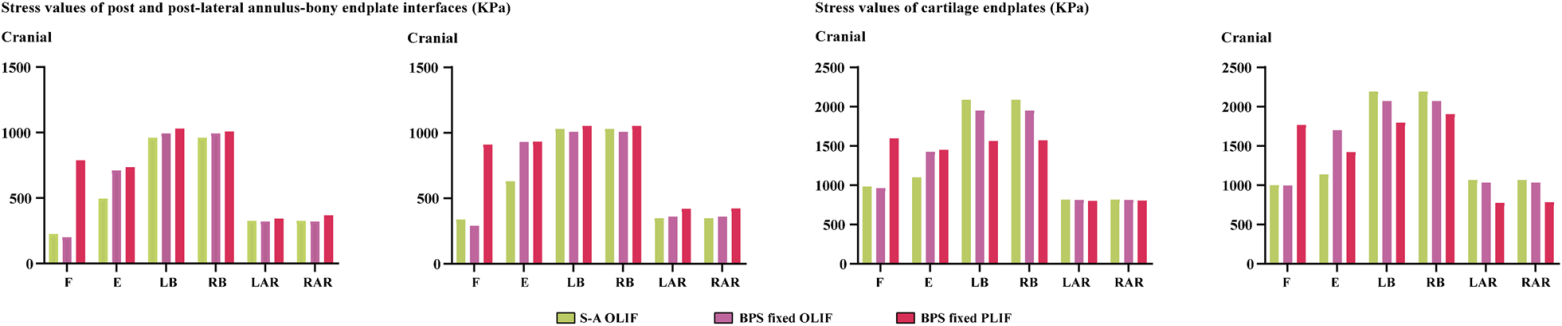
Computational results relate to ASD

**Fig. 4:**
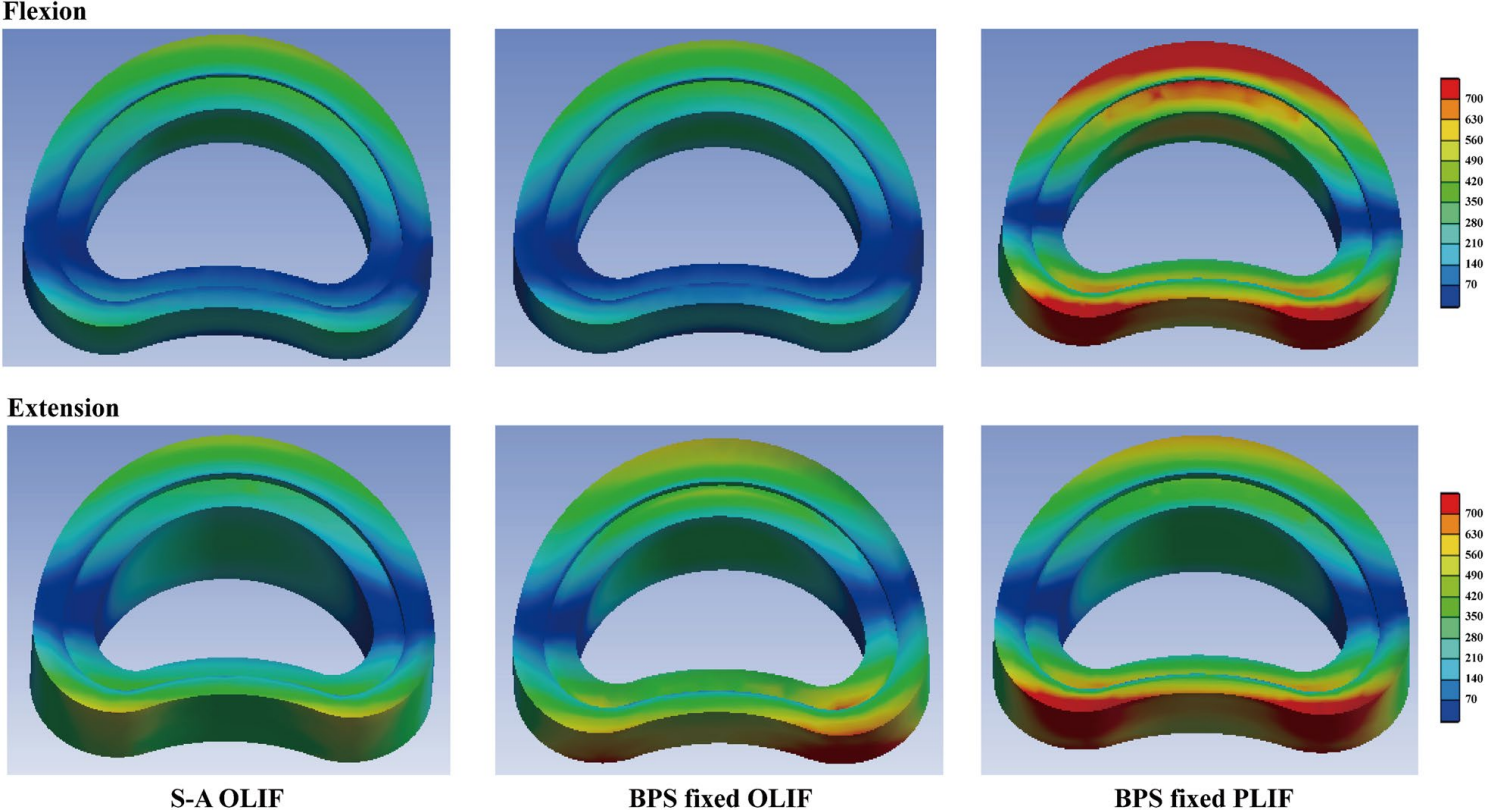
Nephograms of annulus under the flexion and extension loading conditions

In this study, to verify this hypothesis, two different kinds of LIF operations with and without BPS fixation and PLC excision have been simulated in our previously constructed and validated numerical model. Identifying BPS fixation and PLC’s biomechanical significance is of great significance for better understanding the pathological process of ASD generation (Figs. 1,2,3,4).

## Methods

### Model selection, calibration and validation

A three-dimensional finite element model (L3-S1) has been widely used in our previously published studies [23–26]. The model construction, calibration, and validation strategies have been well presented in studies published by co-authors in this study [24, 25, 27]. Specifically, the outline of bony structures reconstructed from CT imaging data was seen as a template, and we constructed smoothed surfaces to cover the reconstructed outlines. This model divided bony structures into cortical, cancellous, and bony endplates (BEPs) [24, 25, 27]. The cortical thickness was 0.8 mm, the thickness of BEPs was defined based on anatomical observation [28], and concave angles and depths of BEPs were also defined based on previously published imaging measurement reports [29, 30]. Ligamentum structures were defined to cable elements in the pre-processing of finite element analysis [25, 26]. Attach points of ligaments were defined based on anatomical observation [25, 26]. Then, non-bony structure models were also constructed, model calibrations were performed based on parameters of intervertebral discs, and calibration and validation processes were presented as follows: To ensuring the computational accuracy of this model, two different calibrated factors, including the average radius ratio of intervertebral disc and the relative position of nucleus, have been calibrated in our published studies. Multi-indicators validation has also been performed in these studies, and the result shows that the current calibrated FE model could good match the real mechanical parameters’ values [31–33]. Recently, different LIF operations with different fixation methods have been simulated in this model, and its corresponding computational results can good explain our clinical observed phenomenon [23, 24, 26]. In a word, based on our published studies, the current model can be directly used in the simulation of LIF operations in study without any necessity of model’s modification. Detailed model construction procedures have been well presented in our published studies.

### LIF simulations with and without BPS fixation and PLC excision

The simulation of oblique lumbar interbody fusion (OLIF) and posterior lumbar interbody fusion (PLIF) operation has been performed based on our literature review and clinical experience [34, 35]. Like our previously published studies, the L4-L5 segment has been selected in the PLIF simulation process, for this motion segment suffers the highest incidence risk of LDDs [34, 35]. In this process, the stand-alone (S-A) OLIF (i.e. OLIF without BPS fixation) and OLIF fixed by BPS have been simulated. In this process, the nucleus, cartilage endplates, and bilateral annulus in regions with OLIF cage insertion were excised, and a 50-mm length OLIF cage was inserted into the interbody space. The axis of the OLIF cage was parallel to the coronal plane of vertebral bodies [23, 24]. When simulating BPS fixed OLIF operation, four cannulated screws were inserted into L4 and L5 vertebral bodies. The axis of the pedicle screws was parallel to that of the pedicle in the transverse and to that of the superior bony endplate on the sagittal plane [32, 34].

In the simulation of traditional PLIF, the osteotomy process was performed as follows. The spinal process of L4 was excised, and the range of bilateral laminectomy was limited to two-thirds lamina on the caudal side. Meanwhile, a medium one-third of the caudal articular process has also been excised [34, 36]. Regarding the excision of soft tissues, PLC in the L4-L5 segment has been excised. Meanwhile, given that the attachment point of L3-L4 plc has been excised, the L3-LE PLC has also been deleted. The ligamentum flavum in the surgical segment, all nucleus, and the post-lateral annulus has also been excised [34]. To simulate the endplate preparation process, all cartilage endplates (CEPs) in both cranial and caudal sides under the outline of annulus excision and nuclectomy have been deleted in PLIF models. To simulate the interbody fusion process, an PLIF cage filled with grafted bone was inserted into the interbody space. During the BPS simulation, four solid pedicle screws were inserted into L4 and L5 vertebral bodies; screw trajectories were kept identical to that of the OLIF model [4, 34, 36].

### Boundary and loading conditions

Consistent with in vitro mechanical tests and our previously published studies, inferior surfaces of LIF models were fixed under six freedom degrees, and all directional moments were applied on the superior surface of L3 to simulate different daily body positions [37, 38]. The mesh size and generation strategy in this study were identical to our study series [23, 24]. The average mesh quality was higher than 0.75 to reduce the incidence rate of mesh deterioration and related computational error. Flexion, extension, left and right lateral bending, and left and right axial rotation were simulated in LIF models. The moment sizes in different body positions were identical to the model validation moment sizes reported by Renner et al. (Flexion: 8Nm, Extension: 6Nm, Bending: 6Nm, Rotation: 4Nm) [39–41]. Given that PLIF models were not symmetrical along the central sagittal plane, both the left and right sides' bending and rotation were computed. By contrast, only unilateral bending and rotation moments were applied in OLIF models for these models were symmetrical along the sagittal plane. By this method, we can increase the computational efficiency in this study.

## Results

To investigate the risk of ASD, biomechanical indicators in the L3-L4 motion segment were computed and recorded. As reported by Adam et al., endplate damage and annulus tear were two main phenotypes of disc degeneration [42, 43]. Therefore, corresponding biomechanical parameter values, including the maximum equivalent stress of both superior and inferior cartilage endplates, and interfaces between post, post-lateral annulus, and bony endplates, were recorded in this study.

There are significant differences in biomechanical parameter values in different body positions. Specifically, in the flexion loading condition, stress values in the model without BPS fixation are even lower than BPS fixed OLIF model. However, higher stress values can be observed in the BPS fixed PLIF model with PLC excision. By contrast, in the extension loading condition, BPS fixed models (including OLIF and PLIF models) present higher stress values than S-A OLIF models. PLC excision only slightly increases stress values in most components. Furthermore, the maximum value of the inferior cartilage endplate is even lower in the PLIF model with PLC excision. By contrast, in the bending loading condition, maximum stress values can be recorded in the S-A OLIF model without BPS fixation and PLC excision in most components, except for superior endplate-annulus interfaces. In the axial rotation loading condition, interfaces stress values were increased, but cartilage endplate stress values were decreased with BPS fixation and PLC excision. Additionally, slightly higher stress values can be recorded on the annulus incision side (i.e. the right side).

## Discussion

In order to investigate whether BPS fixation and PLC excision will deteriorate the local biomechanical environment related to ASD, LIF operations with and without these surgical procedures were simulated in our widely used finite element model. Biomechanical parameters related to two different phenotypes of disc degeneration were recorded. Results show that BPS fixation and PLC excision in the cranial segment will deteriorate the local mechanical environment in different body positions.

The maximum equivalent stress of the interface between bony endplates and annulus and the maximum stress of cartilage endplates were selected as indicators to judge the risk of ASD in this study. Disc degeneration is the main pathological change of ASD; annulus tears and cartilage endplate damage are two main phenotypes of ASD [42, 43]. Specifically, cartilage endplate damage and ossification are the main sources of disc degeneration. Trans-cartilage endplate diffusion is the main routine of nutrition distribution because the blood supply of the intervertebral disc is limited in the out layer of the annulus in adult patients [44, 45]. According to the basic principle of osteogenesis, higher stress value may trigger the ossification of cartilage endplates, resulting in inhibition of the nutrition pathway, accelerating the disc degeneration process, and resulting in a higher risk of ASD [46, 47]. Meanwhile, the higher stress value of cartilage endplates also triggers a higher risk of endplate damage. This also disrupts normal local metabolism, allowing macromolecules to enter the intervertebral disc. Since the nucleus pulposus is a typical antigenic structure, the resulting inflammatory response can also exacerbate disc degeneration and the resulting risk of ASD [47, 48]. Therefore, we believe the computation of the maximum equivalent stress in the cartilage endplate can good represent the risk of ASD.

Meanwhile, the annulus tear is the essential phenotype of disc degeneration in the low lumbar spine. According to the basic principle of materials mechanics, stress concentration can be commonly observed in the interface between two components with significantly different elastic moduli [49, 50]. This was consistent with the phenomenon observed in our clinical practice: the annulus tear was commonly observed in the junction area between bony endplates. Given that stress concentration initially triggers structure failure, we believe the maximum equivalent stress recorded in this interface can represent the risk of annulus tears [47, 51]. The resulting local inflammatory response can also lead to disc degeneration, like cartilage damage in this pathological process [43, 52]. The in-growth of the vessel and nociceptive nerve fibres is the main source of discogenic low back pain for patients with ASD. In conclusion, mechanical parameters recorded in this study can good represent ASD risk in LIF patients.

This study recorded only stress values in the cranial intervertebral disc. That is because ASD commonly occurs in the cranial rather than the caudal motion segment [34, 53]. As mentioned above, LIF and fixation-induced biomechanical deterioration are the initial trigger for ASD. In LIF operations, especially in patients with BPS fixation and PLC excision, the cranial motion segment suffers lower moment arm and results in a poor mechanical environment [54–56]. Consistent with published studies, the biomechanical significance of BPS fixation in the cranial disc has been verified. Given the clinical effect of BPS fixation in constructing instant postoperative stability in the surgical segment, BPS fixation is the gold standard of additional fixation methods for LIF patients. Consistent with this study, removing BPS after credible interbody fusion could alleviate the cranial motion segment’s biomechanical deterioration [21, 24]. However, a second operation is not an acceptable option for all LIF patients. Therefore, we believe the optimization of pedicle screw design and surgical techniques of BPS fixation may be alternative methods to reduce the risk of ASD, but our further clinical studies should verify this hypothesis.

Meanwhile, the excision of the spinal process in the PLIF operation damages the attachment point of PLC in the cranial motion segments [56, 57]. This study proved that this change might trigger a higher risk of ASD by deteriorating the local biomechanical environment. In the PLIF operation, damage to the cranial side spinal process can be effectively avoided. That is because the cranial one-third part of the lamina is not the primary source of nerve decompression for patients with central canal stenosis. The commonly selected laminectomy excluded this part of the lamina. The preservation of the cranial side PLC’s attachment point (i.e. the protection of the cranial part of the spinal process) will not negatively affect the decompression efficiency in the PLIF operation [56, 57]. Therefore, we hypothesize that the protection of the cranial part of PLC reduces the risk of ASD by alleviating biomechanical deterioration. But this should also be verified by clinical evidence.

Indeed, this study still has its inherent limitations. Firstly, clinical follow-up is necessary for the same type studies. FE analysis can only provide a potential variation tendency, which should be verified by clinical evidence. Meanwhile, biological changes cannot be directly simulated in the current FE models. In vitro mechanical tests may have a supplementary effect on FE models. However, limited by the source of fresh specimens, and the interaction between different anatomical parameters cannot be excluded from a small size of fresh specimens in vitro tests, and this experiment has not been performed in this study. In the future studies, additional in vitro tests and clinical follow-up will be performed to recheck the conclusion computed in the current FE LIF models.

## Conclusion

Fixation-induced surgical segment’s high stiffness and the damage of posterior soft tissues together trigger a higher risk of ASD in patients with LIF operations. Optimizing BPS fixation methods and pedicle screw designs and reducing the range of posterior structures excision may be an effective method to reduce the risk of ASD.

## Data Availability

All the data of the manuscript are presented in the paper.
